# Real-World Effectiveness of Baricitinib in Rheumatoid Arthritis: Significant Reduction of Disease Activity Independent of Age in a Multicentre Retrospective Cohort Study

**DOI:** 10.31138/mjr.110525.ain

**Published:** 2026-04-21

**Authors:** Daniele Lini, Matteo Colina, Romina Andracco, Francesca Ometto, Viviana Ravagnani, Alberto Lo Gullo, Simone Parisi, Antonella Farina, Patrizia Del Medico, Marino Paroli, Olga Addimanda, Alessandra Bezzi, Federica Lumetti, Aldo Biagio Molica Colella, Davide Murgia, Maddalena Larosa, Aurora Ianniello, Elena Bravi, Marta Priora, Palma Scolieri, Alessandro Volpe, Francesco Girelli, Elisa Visalli, Alarico Ariani, Eleonora Celletti, Bernd Raffeiner, Veronica Franchina, Rosalba Caccavale, Francesca Serale, Valeria Nucera, Cecilia Giampietro, Riccardo Bixio, Guido Rovera, Alessia Fiorenza, Maria Chiara Ditto, Ilaria Platè, Fabio Mascella, Eleonora Di Donato, Adorni Giuditta, Daniele Santilli, Natalia Mansueto, Gianluca Smerilli, Giulio Ferrero, Amati Gabriele, Francesco Molica Colella, Giorgio Amato, Francesco De Lucia, Ylenia Dal Bosco, Roberta Foti, Simone Bernardi, Myriam Di Penta, Emanuela Sabatini, Francesco Cipollone, Gilda Sandri, Rosario Foti, Gerolamo Bianchi, Maria Cristina Focherini, Vincenzo Bruzzese, Gianluca Lucchini, Eugenio Arrigoni, Rosetta Vitetta, Dario Camellino, Antonio Marchetta, Enrico Fusaro, Massimo Reta, Dilia Giuggioli, Andrea Becciolini

**Affiliations:** 1 Azienda Unità Sanitaria Locale di Bologna -- Policlinico S.Orsola- Azienda Ospedaliera Universitaria-IRCCS di Bologna, Rheumatology Unit, Bologna, Italy;; 2 Rheumatology Service, Section of Internal Medicine. Department of Medicine and Oncology, Ospedale Santa Maria della Scaletta, IMola, Bologna, Italy;; 3 Imperia Hospital, Internal Medicine Unit, Rheumatology Outpatient Clinic, Imperia, Italy;; 4 Azienda ULSS 6 Euganea, Rheumatology Outpatient Clinic, Padova, Italy;; 5 Santa Chiara Hospital APSS Trento, Rheumatology Unit, Trento, Italy;; 6 ARNAS Garibaldi di Catania, Rheumatology Unit, Catania, Italy;; 7 Azienda Ospedaliera Universitaria Città della Salute e della Scienza di Torino, Rheumatology Department, Torino, Italy;; 8 Ospedale “A. Murri” di Fermo, Internal Medicine Unit, Rheumatology Outpatient Clinic, Ferro, Italy;; 9 Civitanova Marche Hospital, Rheumatology outpatient clinic - Internal Medicine Unit, Civitanova Marche, Italy;; 10 Sapienza University of Rome, Polo Pontino, Department of Clinical, Anaesthesiological and Cardiovascular Sciences, Latina, Italy;; 11 ASL Romagna Rimini, Internal Medicine and Rheumatology Unit, Rimini, Italy;; 12 Azienda USL of Modena and University Hospital “Policlinico di Modena”, Rheumatology Unit, Modena, Italy;; 13 Rheumatology Unit, Azienda Ospedaliera Papardo, Messina, Italy;; 14 ASL VC Ospedale S. Andrea, Unit of Rheumatology, Vercelli, Italy;; 15 Azienda Sanitaria Locale 3 Genova, Division of Rheumatology - Medical Specialties Department, Genova, Italy;; 16 ASL Novara, Rheumatology Outpatient Unit, Novara, Italy;; 17 Ospedale G. Da Saliceto, Rheumatology Unit, Piacenza, Italy;; 18 ASL CN1, Rheumatology Day Hospital and Outpatient Clinic, Mondovì, Italy;; 19 “Nuovo Regina Margherita” Hospital, Department of Medical Specialties, Roma, Italy;; 20 IRCCS Sacro Cuore Don Calabria Hospital, Unit of Rheumatology, Negrar, Italy;; 21 Ospedale GB Morgagni - L Pierantoni, Rheumatology Unit, Forlì, Italy;; 22 Policlinico San Marco Hospital, Rheumatology Unit, Catania, Italy;; 23 University Hospital of Parma, Internal Medicine and Rheumatology, Parma, Italy;; 24 Ospedale SS. Annunziata di Chieti, G.d'Annunzio University of Chieti Rheumatology Unit, “Clinica Medica” Institute, Chieti, Italy;; 25 Bolzano Hospital, Rheumatology Unit, Department of Medicine, Bolzano, Italy;; 26 Azienda Ospedaliera Papardo, Medical Direction, Messina, Italy;; 27 University of Modena and Reggio Emilia, Rheumatology Unit, Modena, Italy;; 28 Santa Corona Hospital, Unit of Diagnostic and Interventional Radiology, Pietra Ligure, Italy;; 29 Università Bicocca Milano, Internal Medicine Unit, Milano, Italy

**Keywords:** aging, baricitinib, disease activity, Janus kinase inhibitors, rheumatoid arthritis, treatment outcome

## Abstract

**Objective:**

To evaluate the real-world effectiveness of baricitinib (BARI) in rheumatoid arthritis (RA), compare outcomes in patients <65 years versus ≥65 years, and identify predictors of DAS28-ESR remission/low disease activity (LDA) at 6 and 12 months.

**Methods:**

Retrospective multicentre cohort (n=423). Baseline variables included age, sex, disease duration, and comorbidities. DAS28-ESR was recorded at baseline, 6 and 12 months; remission was defined as DAS28-ESR <2.6 and LDA as ≤3.2. Predictors of remission/LDA were assessed with multivariable logistic regression, reporting odds ratios (ORs), 95% confidence intervals (CIs), and p-values.

**Results:**

Median age was 60 years (IQR 50.5–69.5); 79% were female; median disease duration 76 months (IQR 31–157). Compared with patients <65, those ≥65 had longer disease duration (94 vs 65 months; p<0.05) and more diabetes (16% vs 5%), dyslipidaemia (47% vs 19%), and hypertension (62% vs 33%) (all p<0.05). Median DAS28-ESR fell from 5.42 (IQR 4.84–6.06) at baseline to 3.71 (2.83–4.59) at 6 months and 3.29 (2.42–4.19) at 12 months (global repeated-measures test significant). Remission+LDA was achieved by 135/421 (32.1%) at 6 months and 182/423 (43.0%) at 12 months; remission alone increased from 65/421 (15.4%) to 117/423 (27.7%). Age ≥65 was not associated with response (6 months: OR 0.71, 95% CI 0.44–1.14, p=0.16; 12 months: OR 0.83, 95% CI 0.55–1.24, p=0.37). ACPA positivity independently predicted remission/LDA (6 months: OR 2.32, 95% CI 1.43–3.77, p<0.05; 12 months: OR 1.71, 95% CI 1.12–2.59, p<0.05). Prior JAK inhibitor exposure was not associated with reduced response. Results were consistent in a per-protocol sensitivity analysis.

**Conclusion:**

In this large multicentre real-world cohort, BARI significantly reduced disease activity over 12 months, with comparable effectiveness across age groups. ACPA positivity emerged as an independent predictor of achieving remission/LDA, supporting its potential role in treatment stratification.

## INTRODUCTION

Rheumatoid arthritis (RA) is a chronic autoimmune disease characterised by persistent synovial inflammation and progressive joint destruction, often leading to significant disability and impaired quality of life. Over the past two decades, the treatment landscape of RA has evolved considerably with the introduction of biologic disease-modifying antirheumatic drugs (bDMARDs), which have significantly improved disease outcomes.^1^ More recently, targeted synthetic DMARDs (tsDMARDs), specifically Janus kinase inhibitors (JAKis), have been recognised as an effective and well-tolerated therapeutic class, providing an additional option for patients who fail conventional synthetic DMARDs (csDMARDs).^2,3^

JAKis are small-molecule inhibitors that interfere with intracellular signalling pathways essential for cytokine-mediated immune responses. The Janus kinase (JAK) family comprises four members—JAK1, JAK2, JAK3, and TYK2—each playing a distinct role in RA pathogenesis.^4^ Given their involvement in inflammatory signalling, JAKis have emerged as a viable alternative to bDMARDs, with current EULAR guidelines recommending their use alongside bDMARDs in cases of inadequate response to csDMARD therapy, following appropriate risk assessment.^3^

Baricitinib (BARI), an oral selective JAK1/JAK2 inhibitor, was the first JAKi approved in the European Union (EU) for RA treatment, following evidence from randomised controlled trials (RCTs) demonstrating its superior efficacy over placebo and tumour necrosis factor inhibitors (TNFis) in patients with inadequate response (IR) to csDMARDs, including methotrexate (MTX).^5,6^ Additionally, BARI has shown significant clinical benefits in csDMARD-naïve patients and those with previous inadequate response to bDMARDs.^7,8^ Its approval first took place in Japan, followed by the EU in 2017 and the United States in 2018, and it is now available in over 75 countries.^9–11^

Despite the robust efficacy data from RCTs, real-world evidence is crucial for understanding treatment effectiveness in broader patient populations, as clinical trials often include highly selected participants, limiting generalisability to routine practice settings.^12^ Observational studies provide complementary insights by capturing treatment responses in diverse cohorts, including older adults and patients with multiple comorbidities, who may exhibit different disease courses and drug tolerability.^13,14^

Age-related differences in RA management have gained increasing attention, as older patients frequently present distinct disease phenotypes and a greater burden of comorbidities, which can influence treatment choices and therapeutic outcomes. Although real-world data on tofacitinib are relatively abundant, evidence regarding BARI remains limited, with only a few studies evaluating its long-term effectiveness in clinical practice.^15–20^ A pooled post-hoc analysis of the phase-III RA-BUILD and RA-BEAM trials demonstrated that a 4-mg daily dose of baricitinib achieved ACR20/50/70 response rates that were comparable in patients ≥ 65 years and in younger age groups, with no clinically meaningful increase in serious adverse events (SAEs) versus age-matched placebo controls, indicating that chronological age alone is not a contraindication to JAKi therapy.^21^

Recent real-world observational studies evaluating the clinical response to baricitinib have provided varied insights, particularly regarding age-related differences. Guidelli et al.^17^ reported favourable outcomes in older RA patients despite longer disease duration, whereas Spinelli et al.^18^ emphasised the comparable efficacy of baricitinib across different age groups, consistent with randomised trial data. In contrast, Baldi et al.^16^ observed a lower retention rate in elderly patients, potentially reflecting heightened safety concerns in daily practice. More recent evidence further enriches this picture: Parisi et al.^15^ confirmed baricitinib’s sustained effectiveness up to four years, identifying prior biologic DMARD exposure as a negative predictor of persistence; García-Vivar et al.^22^ demonstrated rapid improvement in disease activity and high drug survival rates in both younger and older adults; and Calvo-García et al.^23^ reported good adherence profiles with substantial corticosteroid-sparing effects across age strata. Given these heterogeneous findings, clarifying age-related differences in treatment outcomes and identifying predictors of response remain important for optimising baricitinib use in clinical practice.

This study aims to evaluate the real-world effectiveness of BARI in RA patients, with a particular focus on age-related differences in treatment response. Specifically, we aim to assess potential differences between younger (<65 years) and older (≥65 years) individuals and identify baseline predictors associated with achieving DAS28-ESR remission or LDA at 6 and 12 months, providing valuable insights into factors influencing treatment success in routine clinical practice.

## MATERIALS AND METHODS

This multicentre retrospective cohort study was conducted across 23 tertiary referral rheumatology centres in Italy as part of the BIRRA (BIologics Retention Rate Assessment) project, which aims to evaluate the long-term retention of innovative antirheumatic therapies. This study was reported following the STROBE statement for observational studies. A completed STROBE checklist is provided as supplementary material. The study received approval from the ethics committees of all participating centres and was conducted in accordance with the Declaration of Helsinki and Good Clinical Practice.^24^ Written informed consent was obtained from all participants prior to inclusion. Patient data were extracted from the clinical databases of each participating centre. The diagnosis of RA was established based on the 2010 ACR/EULAR classification criteria.^25^ All patients were aged ≥18 years and were treated with BARI as monotherapy or in combination with csDMARD, with or without the addition of steroids. The choice between the 2 mg and 4 mg daily doses of baricitinib was based on the treating physician’s clinical judgment, considering factors such as disease activity, patient characteristics, comorbidities, and safety concerns, in accordance with international treatment guidelines and real-world clinical practice.^10,25^ The patient cohort was enrolled between March 2018 and October 2021, with data collection continuing through April 2023, ensuring a minimum follow-up of 18 months for all included patients. Demographic characteristics; smoking habit; previous and ongoing treatments; comorbidities; and laboratory data, including positivity for rheumatoid factor (RF) and anti-citrullinated protein antibody (ACPA), were recorded. Comorbidities considered were diabetes, dyslipidaemia, history of major adverse cardiovascular events (MACE), cancer, and hypertension. Disease activity at baseline, 6 months, and 12 months was assessed in all patients using the Disease Activity Score 28-ESR (DAS28-ESR). In this study, we opted for DAS28-ESR instead of DAS28-CRP, as previous research has shown that DAS28-CRP tends to underestimate disease activity and overestimate improvements in disease status compared to DAS28-ESR.^26^ This choice was made to ensure a more accurate evaluation of disease activity and treatment efficacy.

A cohort of 423 patients initiating BARI was analysed and stratified into two age groups: under 65 years (n=264; 62.4%) and ≥ 65 years (n=159; 37.6%). Baseline characteristics, including demographic data, disease activity, prior treatment history, and comorbidities, were compared between the two groups.

### Statistical Analysis

Descriptive statistics were used for demographic and clinical data. Continuous variables were summarised as medians with interquartile ranges (IQR) and compared between age groups (<65 vs ≥65 years) using the Mann–Whitney U test. Categorical variables were presented as counts (%) and compared using the χ^2^ test or Fisher’s exact test when expected counts were <5. To evaluate within-patient changes in disease activity over time, the Friedman test was applied to DAS28-ESR measured at baseline, 6 months, and 12 months.

For prediction of DAS28-ESR remission or low disease activity (LDA) at 6 and 12 months, we fitted multivariable logistic regression models. Candidate variables were prespecified: age group (≥65 vs <65 years), sex, BMI, disease duration, baseline DAS28-ESR, smoking status (current/former/never), serostatus (RF positivity; ACPA positivity), comorbidities (diabetes, dyslipidaemia, hypertension, prior MACE, cancer), prior exposure to bDMARDs and to tsDMARDs, and concomitant csDMARD use at baseline. Variables with p<0.10 in univariable analyses entered the multivariable model. Results are reported as odds ratios (OR) with 95% confidence intervals (CI).

Analyses followed an intention-to-treat framework including all 423 patients at baseline. No imputation of missing data was performed. For regression models, complete-case analyses were used.

In addition to the primary intention-to-treat (ITT) analysis, we performed a per-protocol (PP) sensitivity analysis at 6 and 12 months. The PP cohort included patients who remained on baricitinib (no recorded suspension) up to the respective timepoint and had a DAS28-ESR assessment available. We repeated descriptive response analyses (remission, LDA, remission+LDA) and the multivariable logistic regression using the same covariate set as in the ITT models. Results are reported as ORs with 95% CIs and qualitatively compared with the ITT estimates.

For all endpoints and descriptive comparisons, variable-specific denominators reflect the number of patients with available data and are reported in the tables and figure legends. All tests were two-sided; p-values <0.05 were considered statistically significant. Analyses were conducted in Jamovi v2.3.21 (www.jamovi.org); figures were produced using Python (Matplotlib, Seaborn).

### Ethics approval

The study was approved by the Comitato Etico dell’Area Vasta Emilia Nord (lead/primary ethics committee; protocol no. 34 713; 28 August 2019) and conducted in accordance with the Declaration of Helsinki and Good Clinical Practice. All participating centres obtained approval from their local Ethics Committees prior to patient inclusion. Written informed consent was obtained from all participants. A full list of site-specific approvals (committee name, protocol number, approval date) is archived by the corresponding author and available upon reasonable request.

**Table 2.** Analysis of predictive factors of DAS28-ESR remission or low-disease activity at 6 months.

**Characteristic**	**Univariate**	**Multivariate**

**≥65 years**	0.67 (0.44–1.04), p 0.07	0.71 (0.44–1.14), p 0.16

**Sex (M vs F)**	0.93 (0.56–1.54), p 0.8	

**BMI**	1.03 (0.96 –1.1), p 0.33	

**Smoke**		
Current vs Ever	0.95 (0.55–1.65), p 0.87	
Former vs Ever	0.9 (0.51–1.61), p 0.74	

**Line of treatment**	0.98 (0.85–1.12), p 0.78	

**Disease duration**	1.00 (0.99–1.00), p 0.61	

**RF** (present)	1.1 (0.71–1.72), p 0.64	

**ACPA** (present)	2.11 (1.33–3.32), p <0.01	2.32 (1.43–3.77), p <0.01

**DM** (present)	0.66 (0.3–1.48), p 0.32	

**Hypertension** (present)	0.8 (0.52–1.23), p 0.32	

**Dyslipidaemia** (present)	0.79 (0.49–1.27), p 0.33	

**Previous MACE**	0.69 (0.28–1.71), p 0.43	

**Previous Neoplasm**	0.99 (0.38–2.5), p 0.99	

**Current csDMARDs**	0.72 (0.46–1.12), p 0.14	

**Current corticosteroid**	1.31 (0.87–1.97), p 0.19	

**Previous JAKi**	0.99 (0.62–1.57), p 0.96	

**Basal DAS28**	0.83 (0.68–1.00), p 0.06	0.78 (0.63–0.97), p 0.03

Odds ratios (OR) with 95% confidence intervals (CI) from logistic regression; p-values from Wald tests.

ACPA: anti-citrullinated protein antibodies; CI: confidence interval; csDMARD: conventional synthetic DMARD; DAS28-ESR: Disease Activity Score (28 joints) using ESR; DM: diabetes mellitus; JAKi: Janus kinase inhibitor; MACE: major adverse cardiovascular events; OR: odds ratio; RF: rheumatoid factor.

**Figure 1.**
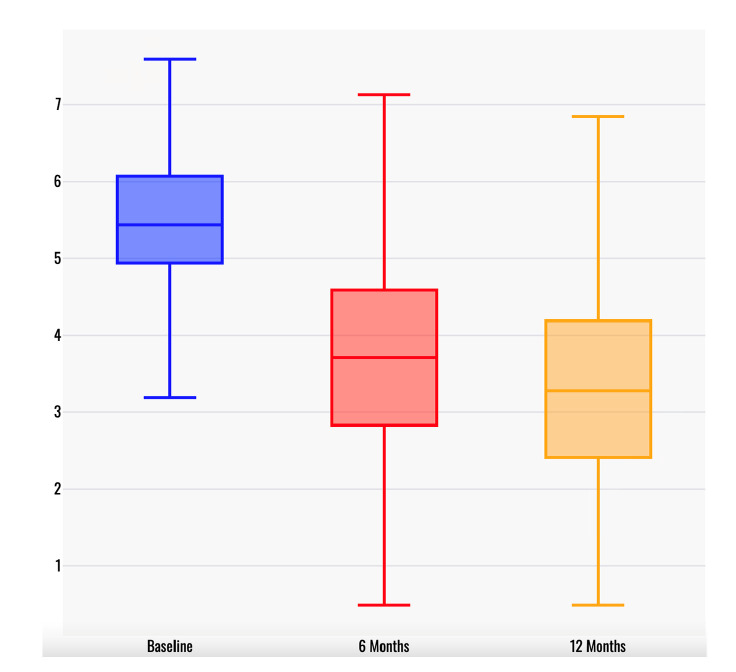
Box plot of DAS28-ESR scores at baseline, 6 months, and 12 months, showing a progressive reduction in disease activity over time.

## RESULTS

### Baseline Characteristics

The baseline characteristics of the study population are summarised in **Table 1**. A total of 423 patients initiated baricitinib. Median age was 60.0 years (IQR 50.5–69.5); 79.4% were female. Median disease duration was 76 months overall, and was longer in patients ≥65 versus <65 years (94 vs 65 months; p<0.05). Comorbidities were more frequent in the older group: diabetes (13.8% vs 4.2%; p<0.05), dyslipidaemia (40.9% vs 17.4%; p<0.05), and hypertension (53.5% vs 29.9%; p<0.05).

**Table 1.** Baseline characteristics of RA patients.

**Characteristic**	**Overall (=423)**	**<65 years (=264)**	**≥65 years (=159)**	**p-value**

**Age, year**, median (IQR)	60 (50.5–69.5)	53.5 (48–59)	72 (68–76)	-

**Sex**, n (%)				
Female	336 (79.4%)	209 (79.2%)	127 (79.9%)	0.86*
Male	87 (20.6%)	55 (20.8%)	32 (20.1%)	

**BMI**, median (IQR) °	24.8 (23–27)	23.6 (22.1–26.2)	25 (23.1–28)	**<0.01** ^§^

**Smoker,** n (%)				
Yes	75/407 (18.4%)	56/252 (22.2%)	19/155 (12.3%)	**<0.01** *
Former	68/407 (16.7%)	26/252 (10.3%)	42/155 (27.1%)	
Ever	264/407 (64.9%)	170/252 (67.5%)	94/155 (60.6%)	

**Disease duration, months**, median (IQR)#	75.5 (31.25–157)	65.0 (29–135.75)	94.5 (41.75–197.25)	**<0.01** ^§^

**RF positive**, n (%)	261/401 (65.1%)	154/251 (61.4%)	107/150 (71.3%)	**0.04** *

**ACPA positive**, n (%)	240/394 (60.9%)	147/249 (59%)	93/145 (64.1%)	0.31*

**DAS28-ESR**, median (IQR)	5.42 (4.84–6.06)	5.37 (4.85–6.05)	5.53 (4.8–6.07)	0.43^§^

**SJC28**, median (IQR)	5 (3–8)	5 (3–8)	6 (2–8)	**0.03** ^§^

**TJC28**, median (IQR)	8 (4–12)	9.5 (5–12)	7 (3–12)	0.69^§^

**Line of treatment, n**, median (IQR)	2 (2–3)	2 (2–3)	2 (2–3)	0.66^§^

**Concomitant csDMARDs**, n (%)				
MTX	125 (29.6%)	72 (27.3%)	53 (33.3%)	
LFN	11(2.6%)	8 (3%)	3 (1.9%)	
HCQ	11 (2.6%)	10 (3.8%)	1 (0.6%)	
SSZ	3 (0.7%)	2 (1.1%)	1 (0.6%)	0.28*
MMF	1 (0.2%)	1 (0.4%)	0	

**Concomitant corticosteroid**, n (%)	204 (48.2%)	133 (50.4%)	71 (44.7%)	0.25*

**Prednisone-equivalent daily dose, mg**, median (IQR)				

**Previous usage of bDMARDs**, n (%)				
Anti-TNF α	256 (60.5%)	145 (54.9%)	111 (69.8%)	**0.01** *
Anti-IL6R	109 (25.8%)	72 (27.3%)	37 (23.3%)	0.51*
IL1Ra	4 (0.9%)	2 (0.8%)	2 (1.3%)	0.60*
CD80/CD86 inhibitor	65 (15.3%)	37 (14%)	28 (17.6%)	0.32*
Anti-CD20	30 (7.1%)	24 (9.1%)	6 (3.8%)	**0.03** *

**Previous usage of tsDMARDs**, n (%)	112 (26.5%)	82 (31.1%)	30 (18.9%)	**<0.01** *

**Comorbidities**, n (%)				
Diabetes	33/379 (8.7%)	11/241 (4.6%)	22/138 (15.9%)	**<0.01** *
Dyslipidaemia	111/379 (29.3%)	46/241 (19.1%)	65/138 (47.1%)	**<0.01** *
Previous MACE	25/378 (6.6%)	9/240 (3.8%)	16/138 (11.6%)	**<0.01** *
Hypertension	164/380 (43.2%)	79/242 (32.6%)	85/138 (61.6%)	**<0.01** *
History of cancer	20/380 (5.3%)	10/242 (4,1%)	10/138 (7.2%)	0.19*

Values are median (IQR). Data missing (overall): ° BMI, n=106; # disease duration, n=5. Statistical tests:

* chi-square;

§ Mann–Whitney U.

ACPA: anti-citrullinated protein antibodies; Anti-IL6R: anti–interleukin-6 receptor; Anti-TNFα: anti–tumour necrosis factor-α; bDMARD: biologic DMARD; BMI: body mass index; csDMARD: conventional synthetic DMARD; DAS28-ESR: Disease Activity Score (28 joints) using ESR; HCQ: hydroxychloroquine; IL1Ra: interleukin-1 receptor antagonist; IQR: interquartile range; JAKi: Janus kinase inhibitor; LFN: leflunomide; MACE: major adverse cardiovascular events; MMF: mycophenolate mofetil; MTX: methotrexate; RF: rheumatoid factor; SJC28: swollen joint count; SSZ: sulfasalazine; TJC28: tender joint count; tsDMARD: targeted synthetic DMARD.

### Disease activity over time

**Figure 2.**
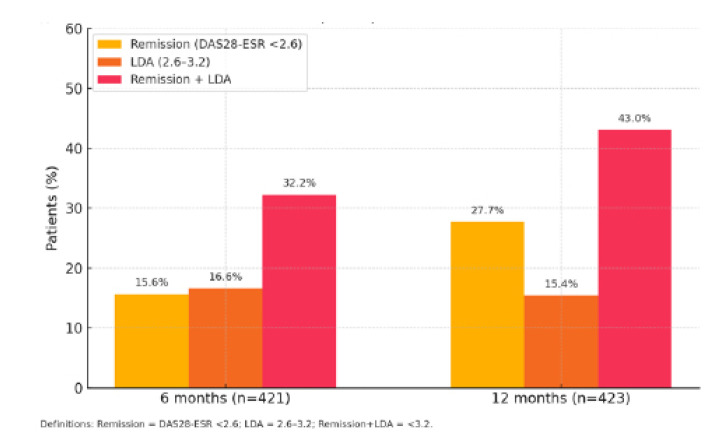
Remission, LDA, and combined remission+LDA at 6 and 12 months in the baricitinib cohort.

DAS28-ESR decreased from 5.42 (IQR 4.84–6.06) at baseline to 3.71 (IQR 2.83–4.59) at 6 months and 3.29 (IQR 2.42–4.19) at 12 months (p<0.05, Friedman test). In the intention-to-treat (ITT) population, remission+L-DA was achieved by 135/421 (32.1%) at 6 months and 182/423 (43.0%) at 12 months. Remission alone increased from 65/421 (15.4%) to 117/423 (27.7%), while LDA without remission accounted for 70/421 (16.6%) and 65/423 (15.4%) at 6 and 12 months, respectively (**Figure 2**).

**Table 3.** Analysis of predictive factors of DAS28-ESR remission or low-disease activity at 12 months.

**Characteristic**	**Univariate**	**Multivariate**

**Over 65 years**	0.83 (0.55–1.24), p 0.37	

**Sex (M vs F)**	0.91 (0.56–1.46), p 0.7	

**BMI**	1.01 (0.94 –1.07), p 0.73	

**Smoke**		
Current vs Ever	0.85 (0.50–1.43), p 0.54	
Former vs Ever	0.94 (0.55–1.62), p 0.84	

**Line of treatment**	0.92 (0.81–1.05), p 0.25	

**Disease duration**	1.00 (0.99–1.00), p 0.84	

**RF** (present)	1.14 (0.75–1.74), p 0.51	

**ACPA** (present)	1.71 (1.12–2.59), p 0.01	

**DM** (present)	1.19 (0.58–2.43), p 0.48	

**Hypertension** (present)	1.25 (0.83–1.88), p 0.27	

**Dyslipidaemia** (present)	1.30 (0.84–2.04), p 0.23	

**Previous MACE**	0.60 (0.26–1.40), p 0.24	

**Previous Neoplasm**	2.14 (0.83–5.50), p 0.11	

**Current csDMARDs**	0.77 (0.51–1.16), p 0.22	

**Current corticosteroid**	1.37 (0.93–2.02), p 0.10	

**Previous JAKi**	0.98 (0.63–1.52), p 0.94	

**Basal DAS28-ESR**	1.08 (0.90–1.30), p 0.39	

Odds ratios (OR) with 95% confidence intervals (CI) from logistic regression; p-values from Wald tests.

ACPA: anti-citrullinated protein antibodies; CI: confidence interval; csDMARD: conventional synthetic DMARD; DAS28-ESR: Disease Activity Score (28 joints) using ESR; DM: diabetes mellitus; JAKi: Janus kinase inhibitor; MACE: major adverse cardiovascular events; OR: odds ratio; RF: rheumatoid factor.

**Figure 3.**
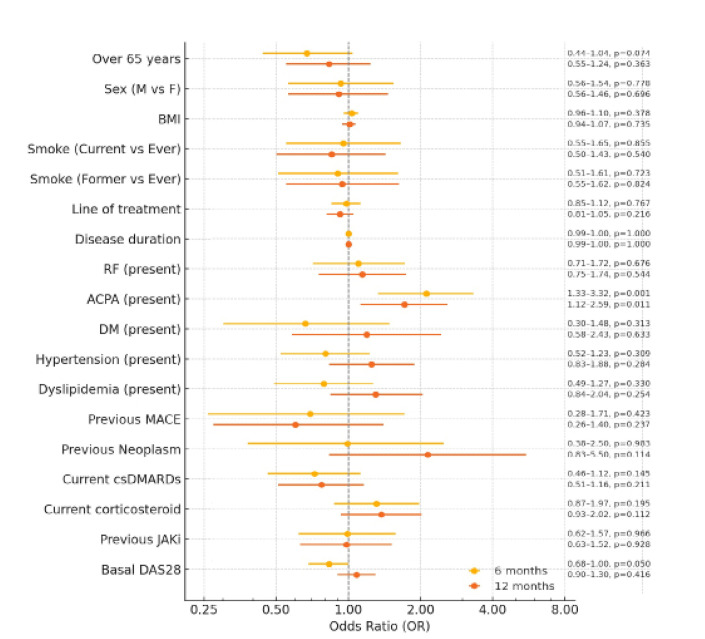
Analysis of predictive factors for DAS28-ESR remission or Low Disease Activity at 6 and 12 months.

### Outcomes by age

At 6 months, remission+LDA occurred in 48/159 (30.2%) in patients ≥65 and 100/262 (38.2%) in those <65 years (chi-square test, p=0.096; Fisher’s exact, p=0.114). At 12 months, rates were 65/159 (40.9%) and 130/263 (49.4%), respectively (chi-square p=0.088; Fisher’s p=0.107). No statistically significant between-group differences were detected at either time point.

### Treatment exposure and per-protocol denominators

Over 12 months, 322/423 (76.1%) had no recorded suspension and 101/423 (23.9%) had a suspension. For per-protocol (PP) analyses (on-drug with DAS28-ESR available at the visit), denominators were 321 at 6 months and 321 at 12 months.

### Per-protocol effectiveness

At 6 months (PP), remission+LDA was 107/321 (33.3%), comprising remission 52/321 (16.2%) and LDA-only 55/321 (17.1%). At 12 months (PP), remission+LDA was 144/321 (44.9%), with remission 92/321 (28.7%) and LDA-only 52/321 (16.2%). PP estimates were concordant in direction and magnitude with ITT results.

### Predictors of remission/LDA

In multivariable ITT models (complete-case), ACPA positivity independently predicted remission/LDA at 6 months (OR 2.76, 95% CI 1.36–5.61; p<0.05) and 12 months (OR 2.35, 95% CI 1.24–4.48; p<0.05). Age ≥65 years was not significant (6 months: OR 0.64, 95% CI 0.35–1.15; p=0.133; 12 months: OR 0.59, 95% CI 0.34–1.02; p=0.059). Prior JAK inhibitor exposure showed no association (6 months: OR 0.77, 95% CI 0.32–1.87; p=0.568; 12 months: OR 0.76, 95% CI 0.33–1.74; p=0.512). Baseline DAS28-ESR was associated with response at 6 months (OR 0.67, 95% CI 0.51–0.86; p<0.05), but not at 12 months (OR 0.90, 95% CI 0.71–1.14; p=0.387).

In multivariable PP models, ACPA positivity remained associated with remission/LDA at 6 months (OR 2.92, 95% CI 1.22–7.02; p<0.05) and 12 months (OR 2.46, 95% CI 1.12–5.39; p<0.05); prior JAK inhibitor exposure remained not associated (6 months: OR 0.82, 95% CI 0.28–2.41; p=0.71; 12 months: OR 0.78, 95% CI 0.28–2.15; p=0.63). Age ≥65 years was not significant (6 months: OR 0.49, 95% CI 0.24–1.01; p=0.052; 12 months: OR 0.60, 95% CI 0.31–1.16; p=0.128). Baseline DAS28-ESR predicted response at 6 months (OR 0.62, 95% CI 0.46–0.85; p<0.05) but not at 12 months (OR 0.93, 95% CI 0.70–1.22; p=0.58).

## DISCUSSION

In this cohort study, we evaluated the clinical response to baricitinib (BARI) in patients with RA, with particular focus on the potential differences between younger (<65 years) and older (≥65 years) individuals and identified baseline predictive factors associated with remission or low disease activity (LDA) at 6 and 12 months.

### Impact of Age on Baricitinib Response

Age is a relevant factor in RA management and may influence treatment response. Although in our study age was not a significant predictor of response to BARI, some clinical differences between age groups warrant consideration. Older patients had a significantly longer disease duration and a higher prior exposure to bDMARDs, particularly anti-TNFα agents, suggesting a more refractory disease history. Additionally, the presence of multiple comorbidities, including hypertension, dyslipidaemia, and diabetes, may have influenced treatment decisions and drug persistence.

Our real-world findings mirror those generated in randomised trials: the proportion of patients achieving DAS28-ESR remission or low disease activity at 6 and 12 months was comparable between individuals < 65 years and those ≥ 65 years, confirming that therapeutic efficacy is preserved in the geriatric population.^21^

Previous studies have indicated that in older patients, JAKi may be less frequently prescribed or discontinued earlier due to safety concerns, particularly regarding cardiovascular and thromboembolic risks.^27^ However, our findings confirm that BARI led to a significant reduction in DAS28-ESR in older patients, despite their higher prior exposure to bDMARDs, suggesting its efficacy even in a more treatment-refractory population. In light of the known increase in cardiovascular and infectious risks associated with JAKi particularly in patients ≥ 65 years with multiple comorbidities,^3,28,29^ older patients receiving BARI should undergo a structured risk assessment and closer clinical follow-up to ensure early detection of adverse events.

Our findings align closely with previous observational studies. For example, Guidelli et al.^17^ observed similar remission/LDA rates at 12 months, supporting our conclusion that baricitinib efficacy does not differ significantly between younger and older RA patients despite differences in baseline characteristics. Conversely, Baldi et al.^16^ observed a higher discontinuation rate among older patients, attributed mainly to safety concerns. More recent reports further substantiate these observations: Parisi et al.^15^ confirmed sustained baricitinib effectiveness over a four-year follow-up, although prior biologic DMARD exposure was a negative predictor of persistence; García-Vivar et al.^22^ found comparable clinical improvements and high drug survival rates across age groups in a large multinational cohort; and Calvo-García et al.^23^ documented substantial corticosteroid-sparing effects in both younger and older adults. These findings, combined with our data, underline the importance of closely monitoring treatment persistence and tolerability in elderly patients receiving baricitinib, while recognising that therapeutic efficacy can be maintained irrespective of age.

#### Impact of ACPA Positivity on Treatment Response

Our multivariable models consistently showed higher odds of remission/LDA in ACPA-positive patients at both 6 and 12 months, in ITT and PP frameworks. These findings align with the hypothesis that ACPA-positive RA represents a distinct disease phenotype characterised by increased immune activation, which may enhance responsiveness to targeted therapies such as JAKi.

Further supporting this hypothesis, a mechanistic study investigating the immunological effects of baricitinib highlighted the role of JAK inhibition in modulating monocyte function. In a pilot investigation, the frequency of circulating monocytes and the IFN signature in monocytes were identified as early markers of response to baricitinib in RA patients. Responders exhibited a higher baseline expression of IFN-related genes and STAT1 phosphorylation, and only these patients showed a significant reduction in circulating monocytes after four weeks of therapy.^30^

This observation is particularly relevant in the context of ACPA-positive RA, as previous research has demonstrated a strong correlation between IFN signature activation and ACPA production. Castañeda Delgado et al. reported that patients with early and established RA exhibit an increased expression of ISG15, an IFN-stimulated gene, compared with early-stage RA patients. Additionally, IFN activation was significantly correlated with ACPA and anti-carbamylated protein antibody positivity, further supporting the notion that IFN-driven immune dysregulation may underlie the distinct pathophysiology of ACPA-positive RA.^31^

However, while our findings align with the hypothesis that ACPA-positive patients respond better to baricitinib, the literature presents conflicting evidence regarding the predictive role of ACPA in JAKi therapy. Takahashi et al.^32^ did not find a significant association between ACPA status and treatment response in a real-world Japanese cohort. Similarly, Harrold et al.^33^ reported that ACPA positivity correlated with a better response to abatacept or rituximab but not to tofacitinib, another JAKi. These discrepancies suggest that the predictive value of ACPA positivity for JAKi remains uncertain and requires further investigation.

Compared with previous publications in the *Mediterranean Journal of Rheumatology* (MJR), this study uniquely explores the predictive role of ACPA positivity for baricitinib treatment outcomes, providing novel real-world insights specific to an aging RA population. To our knowledge, no previous MJR articles have addressed the interaction between serological status and treatment effectiveness of JAK inhibitors in such a large, multicentre cohort, particularly with a focus on elderly patients.

#### Lack of Association with Drug Retention and Long-Term Efficacy

While ACPA positivity was associated with a better clinical response in our study, we did not assess drug survival or long-term treatment retention. Previous research has suggested that seropositivity, particularly the presence of ACPA and RF, may be linked to longer drug survival with certain biologics.^17^ However, in an Italian real-world study on baricitinib, this association was not confirmed, suggesting that baricitinib’s mechanism of action differs from that of drugs targeting B-cell function or ACPA-related pathways.^15^

Moreover, an observational study comparing baricitinib and sarilumab found that both drugs had comparable efficacy over time, raising the possibility that baricitinib may experience a loss of effectiveness in certain patient subgroups in the long run.^34^ Alternatively, the tendency to switch from baricitinib to another therapy might not necessarily indicate a loss of response but could instead reflect the aggressive nature of ACPA-positive RA, which may prompt clinicians to adjust treatment strategies to achieve better disease control.

Another potential explanation is the evolving regulatory landscape and safety concerns associated with JAK inhibitors, which may have influenced treatment retention. Clinicians might be more cautious in continuing JAKi therapy in certain patients, particularly those at higher risk for adverse events, leading to earlier treatment discontinuation in some cases. These factors should be considered in future studies assessing the long-term persistence of JAKi in real-world settings.

#### Sustained Reduction in Disease Activity

Despite these considerations, our study demonstrated a significant and sustained reduction in disease activity over time. The median DAS28-ESR decreased from 5.42 at baseline to 3.29 at 12 months (p<0.05), with a steady increase in the proportion of patients achieving remission or LDA. These findings further reinforce the role of baricitinib as an effective treatment option for RA, consistent with long-term extension studies showing its sustained efficacy over five years.^35^

#### JAK inhibitor cycling

In our cohort, prior JAK inhibitor exposure was not associated with reduced odds of achieving remission/LDA (adjusted ORs ≈0.8 at 6 and 12 months; both p≥0.05), consistent with registry data supporting the feasibility of JAK-to-JAK cycling.^36,37^ Nonetheless, when the first JAKi was discontinued for adverse events, subsequent response may be attenuated, warranting individualised decisions.^36^

Findings were robust across analytic populations. Per-protocol estimates of remission/LDA at 6 and 12 months (33.3% and 44.9%, respectively) closely matched the ITT results (32.1% and 43.0%), and the pattern of independent predictors was unchanged: ACPA positivity remained a significant predictor at both timepoints, while prior JAK inhibitor exposure did not reduce the odds of response. Because PP analyses preferentially include treatment-persistent patients, these results should be interpreted alongside the ITT estimates.

#### Limitations

This retrospective, multicentre design entails potential selection and information biases and residual confounding by indication, as treatment allocation was not randomised. We did not include an active comparator nor apply balancing methods (e.g., propensity scores); time-varying exposures during follow-up (changes in concomitant csDMARDs or corticosteroids and baricitinib dose adjustments) were not modelled. Outcomes relied on DAS28-ESR only; because ESR is influenced by age and comorbidities, results were not cross-validated with CDAI/SDAI or DAS28-CRP. Standardised PROs and radiographic progression were not collected across centres. Corticosteroid dosing/tapering was not standardised, limiting assessment of steroid-sparing effects, and adverse events were not systematically captured, precluding a benefit–risk appraisal. The impact of baricitinib dose (2 mg vs 4 mg) and dose modifications was not analysed. Missing data were handled with complete-case analyses, which may bias estimates if data were not missing at random. We did not perform a formal drug-survival analysis nor external validation of the prediction model, and generalisability may be limited to tertiary referral centres in Italy.

## CONCLUSIONS

In this large multicentre real-world cohort, baricitinib produced sustained reductions in DAS28-ESR at 6 and 12 months. Efficacy was comparable in patients ≥65 and <65 years despite a heavier comorbidity and treatment burden in older adults. ACPA positivity emerged as the only consistent independent predictor of achieving remission or low disease activity at both time points, whereas other demographic and clinical features, including prior JAK inhibitor exposure, were not associated with response in multivariable models. Taken together, these findings support baricitinib as a viable option across age groups, with individualised risk assessment and close monitoring particularly in elderly patients with comorbidities. Prospective studies with standardised patient-reported outcomes, steroid-sparing endpoints, dose–response analyses (2 mg vs 4 mg), drug survival, and external validation are warranted to confirm the predictive role of ACPA and to refine sequencing strategies (including JAK-to-JAK cycling) in routine practice.

## Data Availability

Individual-level data cannot be shared publicly due to privacy regulations; de-identified data and the analysis code used for the main tables/figures are available from the corresponding author upon reasonable request.

## CONGRESS ABSTRACT PRESENTATION

Preliminary data from this study have not been presented at any scientific meeting or published as a congress abstract.
